# Impulse Inhibition Ability With Methamphetamine Dependents Varies at Different Abstinence Stages

**DOI:** 10.3389/fpsyt.2021.626535

**Published:** 2021-02-19

**Authors:** Weijun Liu, Yu Tian, Xinyu Yan, Jiemin Yang

**Affiliations:** ^1^Institute of Brain and Psychological Sciences, Sichuan Normal University, Chengdu, China; ^2^Faculty of Psychology, Southwest University, Chongqing, China

**Keywords:** behavioral inhibition, impulsivity, methamphetamine, two-choice oddball, abstinence duration

## Abstract

**Objective:** The purpose of this study is to evaluate whether the impulse inhibition ability with methamphetamine dependents would vary at different abstinence stages.

**Methods:** Sixty-three methamphetamine dependents, including 31 short-term (< 10 months) and 32 long-term (≥ 10 months) abstinence participants, were recruited for this study. In addition, 33 men were recruited as the healthy control (HC) group. All participants performed a two-choice oddball task, which is well-established to assess impulse inhibition. Accuracy for deviant trials and deviant–standard reaction time (RT) delay were computed as indexes of impulse inhibition.

**Results:** The accuracy for deviant trials was significantly decreased in short-term abstinence subjects (90.61%) compared to HC subjects (95.42%, *p* < 0.01), which was coupled with a shorter RT delay reflecting greater impulsivity in the short-term group vs. the HC group (47 vs. 73 ms, *p* < 0.01). However, impulse inhibition was improved in the long-term group, shown by the increased accuracy for deviant trials in the long-term group compared to the short-term group (94.28 vs. 90.61%, *p* < 0.05) and the similar accuracy for the long-term and HC groups (*p* > 0.05). Further regression analyses confirmed that the abstinence duration positively predicted impulse inhibition of methamphetamine dependents, both in accuracy and RT for deviant stimulus (β = 0.294, *p* = 0.019; β = 0.337, *p* = 0.007).

**Conclusion:** These results suggest that long-term abstinence is more effective in improving impulse inhibition with methamphetamine dependents.

## Introduction

Impulsivity is defined as a predisposition toward rapid, unplanned reactions to internal, or external stimuli regardless of the potential negative consequences of these reactions (1–3). Impulsivity is associated with an increased likelihood of addiction (4), attention deficit hyperactivity disorder (5), and even antisocial behavior (6). Therefore, the ability to inhibit impulses is extremely important for humans.

Methamphetamine (MA) dependence induces impulsivity (7) and causes cognitive function decline (8). Previous research studies have investigated the negative impact of MA use in the Stop-signal task and Stroop task, showing a longer stop-signal RT (9) and higher error rate (10) in MA dependents. On the other hand, the MA dependent showed significantly worse performance on a test of processing speed (11). Moreover, evidence has shown that 56% of MA users have engaged in aggressive behaviors in social situations (12). These studies indicate that MA dependents have an impulse inhibition deficit.

Several studies have shown neural abnormalities in MA dependents, which can serve to promote impulsivity in MA dependents. One study showed that the basal ganglia of MA dependents were different from those of healthy humans; for example, they showed increased extracellular dopamine concentrations and reduced availability of dopamine transporter (DAT) (13). In addition, MA dependents were found to exhibit abnormal functional connectivity in the corticostriatal circuits, and the resting functional connectivity of the midbrain with the prefrontal cortex (PFC) in abstinent MA dependents was stronger than that in HC subjects (14). Interestingly, one study showed that no difference between long-term abstinent MA dependents and HCs in Stroop RT interference (15). In addition, one study found that a 2-week period of withdrawal improved the right DAT binding and the executive control in MA dependents, suggesting an improvement of prefrontal cognitive control function after abstinence (16). Based on the evidence above, we hypothesized that long-term abstinence (i.e., 10 months or longer) may improve impulse inhibition in MA dependents. To test this hypothesis, the current study investigated the differences of impulse inhibition across different abstinence stages (short-term and long-term) in MA dependents using the two-choice oddball task.

## Methods

### Participants

Sixty-three MA dependents who met the *Diagnostic and Statistical Manual of Mental Disorders (5th edition)* criteria from Da Lian Shan Addiction Rehabilitation Center were recruited for the current study. They were received compulsory drug treatment when they were detected by police for last drug use, and they have no chance to use drug in the next 2 years. The eligibility criteria included no use of drugs other than MA, no physical disability, and no acute physical or psychiatric illness. The exclusion criteria included the use of multiple drugs, current medical conditions and medication use, and the receipt of brain stimulation therapy (Transcranial Direct Current Stimulation or Transcranial Magnetic Stimulation). To balance the sample size between short- and long-term groups, we designated 31 participants who had abstinence periods < 10 months [mean ± SD, [6.17 ± 2.60] months, median 7 months, range 0.17–9 months] as the MA-short group; 32 participants who had abstinence durations ≥ 10 months [mean ± SD, [13.13 ± 1.93] months, median 13.17 months, range 10–17.17 months] served as the MA-long group. The two groups were matched on variables related to history of MA use, such as maximum intake (*p* > 0.05), average intake weekly (*p* > 0.05), and years of MA use before abstinence (*p* > 0.05).

In addition, 33 healthy male participants were recruited as the HC group. The three groups were matched on demographic variables, such as age (*p* > 0.05), education (*p* > 0.05), alcohol use (*p* > 0.05), smoking habits (*p* > 0.05), neuroticism (*p* > 0.05), and Barratt Impulsiveness Scale scores (*p* > 0.05) (Table 1).

**Table 1:** Demographic Data (M ± SD) of the Healthy Control Group, MA-Short, and MA-Long Abstinence Group.

	**HC (*n =* 33)**	**MA-short (*n =* 31)**	**MA-long (*n =* 32)**	***F/χ^2^/t***	***P***
Sex	Male	Male	Male	NA	NA
Age (years)	35.15 ± 9.69	39.03 ± 5.90	37.47 ± 8.10	1.87	0.16
BIS-11	57.06 ± 8.45	60.52 ± 6.69	59.88 ± 7.00	2.00	0.14
Neuroticism (Big Five Personality Inventory)	31.53 ± 8.52	32.16 ± 7.10	32.81 ± 7.13	0.17	0.85
Smoking (%)	84.80	90.30	90.60	0.68	0.71
Alcohol use (%)	66.70	48.40	71.90	4.08	0.13
Education	3.12 ±.89	2.48 ± 1.15	2.87 ± 1.00	9.11	0.33
Maximum intake (g)	NA	0.70 ± 0.46	0.63 ± 0.34	0.71	0.48
Average intake weekly	NA	2.31 ± 2.02	2.74 ± 1.90	−0.86	0.39
Use duration (years)	NA	6.81 ± 3.37	5.93 ± 2.73	1.13	0.27

*Unit for education: denoted as 1 for primary school, educated for 6 years; 2 for junior high school, educated for 9 years; 3 for senior high school, educated for 12 years; 4 for college, educated for 16 years; 5 for post-graduate, educated for 19 years. NA, means not available*.

All participants were right-handed and had normal or corrected-to-normal vision. All the participants participated in the study voluntarily and gave written informed consent. The study was approved by the local ethics committee of human research at Sichuan Normal University and Southwest University in China. The experimental procedures followed the ethical principles of the 1964 Declaration of Helsinki.

### Behavioral Task

We used the two-choice oddball task to examine impulse inhibition, which involved the response to the standard stimulus involves habitual response while the response to the deviant stimulus involves inhibition of habitual motor response and the generation of a different motor response. This task provides both accuracy and response time as indicators of impulse inhibition (17–19).

In the two-choice oddball task, each trial started with a jittered fixation cross, varying from 500 to 1,500 ms. Following this, the task stimulus was presented. For one-half of the participants in each treatment group, if the standard stimulus (“W”; 80% of trials) was presented, they were to press “F” with their left index finger as quickly as possible. If the task stimulus was the deviant stimulus (“M”; 20% of trials), they were to press the “J” key with their right index finger. For the second half of the participants, the response keys were reversed (i.e., they were to press “J” for standard stimuli and “F” for deviant stimuli). Before the formal task, each participant had completed 15 practice trials to familiarize them with the procedure. To avoid the practice effect, the formal experiment did not start until participants had achieved 100% accuracy for both standard and deviant stimuli during practice. At the end of the experiment, participants were told their accuracy as feedback on their performance. Behavioral impulsivity was primarily indicated by the levels of accuracy reduction (i.e., standard minus deviant stimuli accuracy, ACC cost), during deviant vs. standard trials (18). The deviant minus standard RT delay was also recorded to provide context for how ACC cost was altered.

### Statistical Analysis

Where appropriate, one-way analysis of variance (ANOVA), student's *t-*test, and chi-square test were used to compare the differences in demographic variables among the HC, short-term abstinence, and long-term abstinence groups. To analyze the impulse inhibition of participants in the two MA dependence groups compared with that of the HC group, the Bonferroni comparison was used for *post-hoc* comparisons after a statistically significant one-way ANOVA effect appeared. Linear regression was computed with duration of abstinence as predictor and accuracy for deviant stimulus, RT for deviant stimulus, ACC cost, or RT delay as the outcome, respectively. All statistical analyses were performed with SPSS 22.0 (IBM, Armonk, NY).

## Results

The three groups had significant differences in the accuracy of deviant trials [F_(2,93)_ = 6.22, *p* < 0.01] and in RT delay [F_(2,93)_ = 6.67, *p* < 0.01] and ACC cost [F_(2,93)_ = 6.21, *p* < 0.01].

*Post-hoc* pairwise comparisons showed lower accuracy for deviant trials in the MA-short group (90.61%) compared to the HC group (95.42%, *p* < 0.01), indicating enhanced impulsivity in the former group. In addition, the MA-short group showed a shorter RT delay compared to the HC group (MA-short vs. HC, 47 vs. 73 ms, *p* < 0.01), suggesting the fast response tendencies of the MA-short group at the expense of the goal (correct response). However, the MA-long group (M = 94.28%) exhibited significantly higher accuracy during deviant trials compared to the MA-short group (M = 90.61%; *p* < 0.05), which was confirmed by the analysis of ACC cost (4.10 vs. 7.80%, *p* < 0.05). In addition, the MA-long group and HC group showed no significant differences in accuracy of deviant trials, ACC cost, or RT delay.

**Figure 1:**
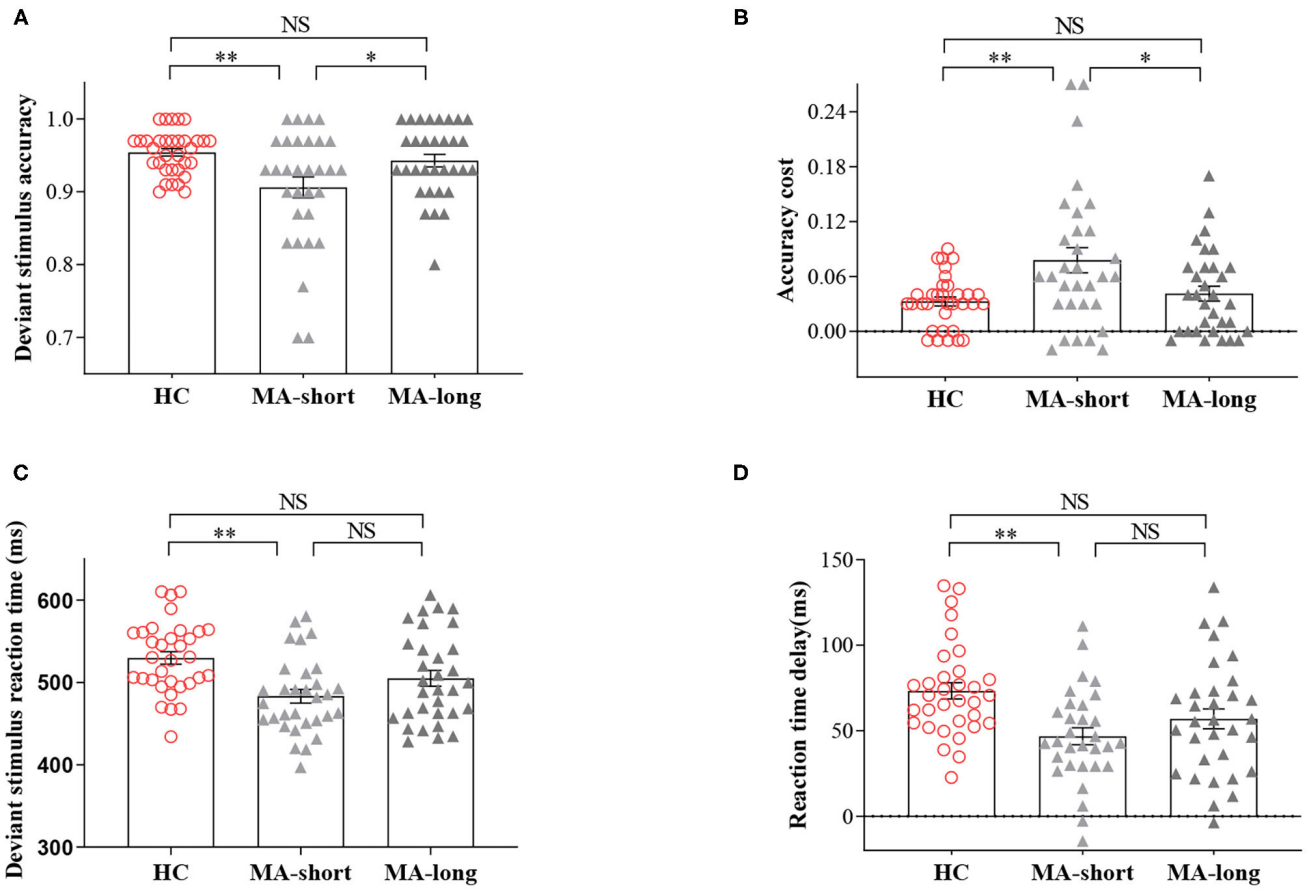
Findings of the two-choice oddball task. **(A)** The accuracy of deviant trials across the short-abstinent, long-abstinent, and healthy control groups. **(B)** The comparisons of standard-deviant ACC cost across the three groups. **(C)** The reaction time of deviant trials across the three groups. **(D)** The comparisons of deviant -standard RT delay across the three groups. NS, indicates not statistically. *p < 0.05; **p < 0.01. Bars denote means, error bars denote standard errors of the mean, circles, and triangles denote individual data points.

It is worth noting that the MA-long group exhibited an RT delay similar to that of the MA-short group, suggesting that the accuracy improvement in the MA-long group was not at the expense of a greater RT delay (Figure 1).

### Regression Analyses

**Figure 2:**
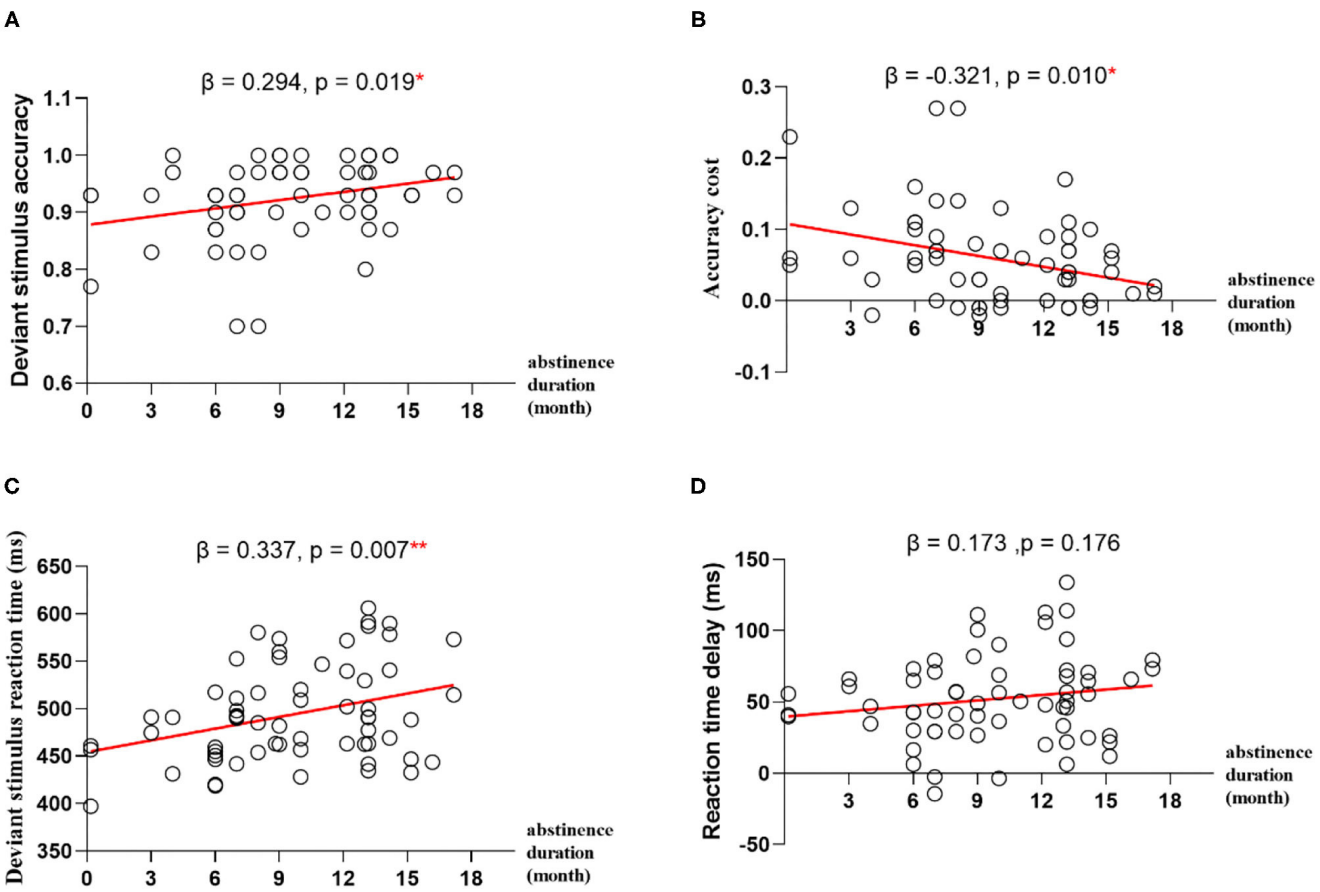
Regression analyses between abstinence duration and impulse inhibition. **(A)** The abstinence duration predicts deviant stimulus accuracy. **(B)** The abstinence duration predicts deviant stimulus accuracy cost. **(C)** The abstinence duration predicts deviant stimulus reaction time. **(D)** The abstinence duration predicts deviant stimulus reaction time delay. *p < 0.05, **p < 0.01.

To explore whether abstinence duration can predict the performance of impulse inhibition, we computed linear regression with duration of abstinence as predictor and accuracy for deviant stimulus, RT for deviant stimulus, ACC cost, or RT delay as outcome, respectively. The results showed that the abstinence duration positively predicted deviant stimulus ACCand RT (β = 0.294, *p* = 0.019, R^2^ = 0.087; β = 0.337, *p* = 0.007, R^2^ = 0.113), and the abstinence duration negatively predict ACC cost (β = −0.321, *p* = 0.010, R^2^ = 0.103) (Figure 2).

## Discussion

Our study demonstrated that impulse inhibition improved with long-term MA abstinence, and abstinence duration can effectively predict the impulse inhibition with MA dependents. On the one hand, our study showed that impulse inhibition deficits in naturally abstinent MA users lasted for about 10 months after abstinence, and individuals in the MA-short abstinence group exhibited lower response accuracy and a shorter RT delay compared to the HC group. However, when the duration of abstinence lasted for more than 10 months, impulse inhibition improved to levels similar to those of the HC group, and the accuracy improvement was not at the expense of a greater RT delay. On the other hand, we also observed that the abstinence duration positively predicted impulse inhibition of MA dependents, both in ACC and RT for deviant stimulus.

Increasing evidence suggests the cognitive capacity and brain functions of the long-term abstinence can be improved in MA users (20). A longitudinal positron emission tomography study documented the improvement of striatal DAT loss in MA dependents within 12–17 months of abstinence (21) and the improvement of thalamic metabolism within a mean of 14 months of abstinence. In contrast, neither of these improvements were observed in short-term abstinence (22). However, none of the prior studies have directly examined what the abstinence duration affects MA-induced differences of impulse inhibition. Here, our study demonstrated that the MA-short abstinence group exhibited worse performance on the two-choice oddball task than the HC group, while these performances were not observed after 10 months of abstinence. These results suggest that it is important to provide more intervention resources to patients in the early stage of rehabilitation than the late stage, particularly concerning the reduction of impulsive drug reuse (23).

This study has several limitations. First, we only included male MA dependents because males were more likely to use MA. Considering the differences in behavioral impulsivity related to drug use between men and women (24), women should be recruited in future research. Second, this study used a cross-sectional approach, and the neural plasticity mechanisms supporting the improved impulse inhibition over time were unknown. Thus, future investigations employing longitudinal neural imaging are necessary.

In summary, the present results suggest that behavioral inhibition deficits persist for about 10 months after MA abstinence. Long-term abstinence beyond 10 months may improve the patients' impulse inhibition to a level similar to that of the healthy population.

## Data Availability Statement

The raw data supporting the conclusions of this article will be made available by the authors, without undue reservation.

## Ethics Statement

The studies involving human participants were reviewed and approved by Human Subjects Ethics Sub-committee of the Sichuan Normal University in China. The patients/participants provided their written informed consent to participate in this study.

## Author Contributions

WL, YT, and JY conceived and designed the study. WL data collection and statistical analyses. WL and JY data interpretation. WL, YT, XY, and JY wrote the final manuscript. All authors contributed to reviewed, and approved the final manuscript.

## Conflict of Interest

The authors declare that the research was conducted in the absence of any commercial or financial relationships that could be construed as a potential conflict of interest.

## References

[1] Moeller FG Barratt ES Dougherty DM Schmitz JM Swann AC. Psychiatric aspects of impulsivity. Am J Psychiatry. (2001) 158:1783–93. 10.1176/appi.ajp.158.11.1783 11691682

[2] Campbell RJ. Psychiatric dictionary, 6th ed. New York, NY: Oxford University Press (1989).

[3] Dickman SJ. Impulsivity and information processing. In: McCown WG Johnson JL Shure MB editors. The Impulsive Client: Theory, Research, and Treatment. Washington, DC: American Psychological Association (1993). p. 151–84. 10.1037/10500-010

[4] Kale D Stautz K Cooper A. Impulsivity related personality traits and cigarette smoking in adults: a meta-analysis using the UPPS-P model of impulsivity and reward sensitivity. Drug Alcohol Depend. (2018) 185:149–67. 10.1016/j.drugalcdep.2018.01.003 29453142

[5] Winstanley CA Eagle DM Robbins TW. Behavioral models of impulsivity in relation to ADHD: translation between clinical and preclinical studies. Clin Psychol Rev. (2006) 26:379–95. 10.1016/j.cpr.2006.01.001 16504359 PMC1892795

[6] Mann FD Engelhardt L Briley DA Grotzinger AD Patterson MW Tackett JL. Sensation seeking and impulsive traits as personality endophenotypes for antisocial behavior: evidence from two independent samples. Pers Individ Dif. (2017) 105:30–9. 10.1016/j.paid.2016.09.018 28824215 PMC5560504

[7] Jones HW Dean AC Price KA London ED. Increased self-reported impulsivity in methamphetamine users maintaining drug abstinence. Am J Drug Alcohol Abuse. (2016) 42:500–6. 10.1080/00952990.2016.1192639 27398730 PMC5055455

[8] Dean AC Groman SM Morales AM London ED. An evaluation of the evidence that methamphetamine abuse causes cognitive decline in humans. Neuropsychopharmacology. (2013) 38:259–74. 10.1038/npp.2012.179 22948978 PMC3527116

[9] Monterosso JR Aron AR Cordova X Xu J London ED. Deficits in response inhibition associated with chronic methamphetamine abuse. Drug Alcohol Depend. (2005) 79:273–7. 10.1016/j.drugalcdep.2005.02.002 15967595

[10] Nestor LJ Ghahremani DG Monterosso J London ED. Prefrontal hypoactivation during cognitive control in early abstinent methamphetamine-dependent subjects. Psychiatry Res. (2011) 194:287–95. 10.1016/j.pscychresns.2011.04.010 22047731 PMC3225642

[11] Simon SL Dean AC Cordova X Monterosso JR London ED. Methamphetamine dependence and neuropsychological functioning: evaluating change during early abstinence. J Stud Alcohol Drugs. (2010) 71:335–44. 10.15288/jsad.2010.71.335 20409426 PMC2859784

[12] Brecht ML Herbeck D. Methamphetamine use and violent behavior: user perceptions and predictors. J Drug Issues. (2013) 43:468–82. 10.1177/0022042613491098 26594058 PMC4651438

[13] London ED Kohno M Morales AM Ballard ME. Chronic methamphetamine abuse and corticostriatal deficits revealed by neuroimaging. Brain Res. (2015) 1628:174–85. 10.1016/j.brainres.2014.10.044 25451127 PMC4418947

[14] Kohno M Morales AM Ghahremani DG Hellemann G London ED. Risky decision making, prefrontal cortex, and mesocorticolimbic functional connectivity in methamphetamine dependence. JAMA Psychiatry. (2014) 71:812–20. 10.1001/jamapsychiatry.2014.399 24850532 PMC4119006

[15] Salo R Nordahl TE Galloway GP Moore CD Waters C Leamon MH. Drug abstinence and cognitive control in methamphetamine-dependent individuals. J Subst Abuse Treat. (2009) 37:292–7. 10.1016/j.jsat.2009.03.004 19339145 PMC2739270

[16] Chou YH Huang WS Su TP Lu RB Wan FJ Fu YK. Dopamine transporters and cognitive function in methamphetamine abuser after a short abstinence: a SPECT study. Eur Neuropsychopharmacol. (2007) 17:46–52. 10.1016/j.euroneuro.2006.05.002 16842981

[17] Yuan J He Y Qinglin Z Chen A Li H. Gender differences in behavioral inhibitory control: ERP evidence from a two-choice oddball task. Psychophysiology. (2008) 45:986–93. 10.1111/j.1469-8986.2008.00693.x 18778319

[18] Yuan J Xu M Yang J Li H. The application of the two-choice oddball paradigm to the research of behavioral inhibitory control. Sci Sin Vitae. (2017) 47:1065–73. 10.1360/N052017-00125

[19] Xin Z Ting LX Yi ZX Li D Bao ZA. Response inhibition of cigarette-related cues in male light smokers: behavioral evidence using a two-choice oddball paradigm. Front Psychol. (2015) 6:1506. 10.3389/fpsyg.2015.01506 26528200 PMC4606050

[20] Hart CL Marvin CB Silver R Smith EE. Is cognitive functioning impaired in methamphetamine users? A critical review. Neuropsychopharmacology. (2012) 37:586–608. 10.1038/npp.2011.276 22089317 PMC3260986

[21] Volkow ND Linda C Gene-Jack W Fowler JS Dinko F Mark S. Loss of dopamine transporters in methamphetamine abusers recovers with protracted abstinence. J Neurosci. (2001) 21:9414–8. 10.1523/JNEUROSCI.21-23-09414.2001 11717374 PMC6763886

[22] Wang GJ Volkow ND Chang L Miller E Sedler M Hitzemann R. Partial recovery of brain metabolism in methamphetamine abusers after protracted abstinence. Am J Psychiatry. (2004) 161:242–8. 10.1176/appi.ajp.161.2.242 14754772

[23] Yuan J Liu W Liang Q Cao X Lucas MV Yuan TF. Effect of low-frequency repetitive transcranial magnetic stimulation on impulse inhibition in abstinent patients with methamphetamine addiction: a randomized clinical trial. JAMA Network Open. (2020) 3:e200910. 10.1001/jamanetworkopen.2020.0910 32167568 PMC7070234

[24] Scheffer M Almeida RMMD. Alcohol consumption and differences between men and women: impulsive behavior, cognitive and neurochemistry aspects. Neuropsicol Lat. (2010). 2:1–11. Available online at: http://pepsic.bvsalud.org/pdf/rnl/v2n3/v2n3a01.pdf

